# Condition Dependent Effects on Sex Allocation and Reproductive Effort in Sequential Hermaphrodites

**DOI:** 10.1371/journal.pone.0109626

**Published:** 2014-10-10

**Authors:** Lock Rogers, Alan Koch

**Affiliations:** 1 Department of Biology, Agnes Scott College, Decatur, Georgia, United States of America; 2 Department of Mathematics, Agnes Scott College, Decatur, Georgia, United States of America; UC Santa Barbara, United States of America

## Abstract

Theory predicts the optimal timing of sex change will be the age or size at which half of an individual's expected fitness comes through reproduction as a male and half through reproduction as a female. In this way, sex allocation across the lifetime of a sequential hermaphrodite parallels the sex allocation of an outbreeding species exhibiting a 1∶1 ratio of sons to daughters. However, the expectation of a 1∶1 sex ratio is sensitive to variation in individual condition. If individuals within a population vary in condition, high-condition individuals are predicted to make increased allocations to the sex with the higher variance in reproductive success. An oft-cited example of this effect is seen in red deer, *Cervus elaphus*, in which mothers of high condition are more likely to produce sons, while those in low condition are more likely to produce daughters. Here, we show that individual condition is predicted to similarly affect the pattern of sex allocation, and thus the allocation of reproductive effort, in sequential hermaphrodites. High-condition sex-changers are expected to obtain more than half of their fitness in the high-payoff second sex and, as a result, are expected to reduce the allocation of reproductive effort in the initial sex. While the sex ratio in populations of sequential hermaphrodites is always skewed towards an excess of the initial sex, condition dependence is predicted to increase this effect.

## Introduction

**Figure 1:**
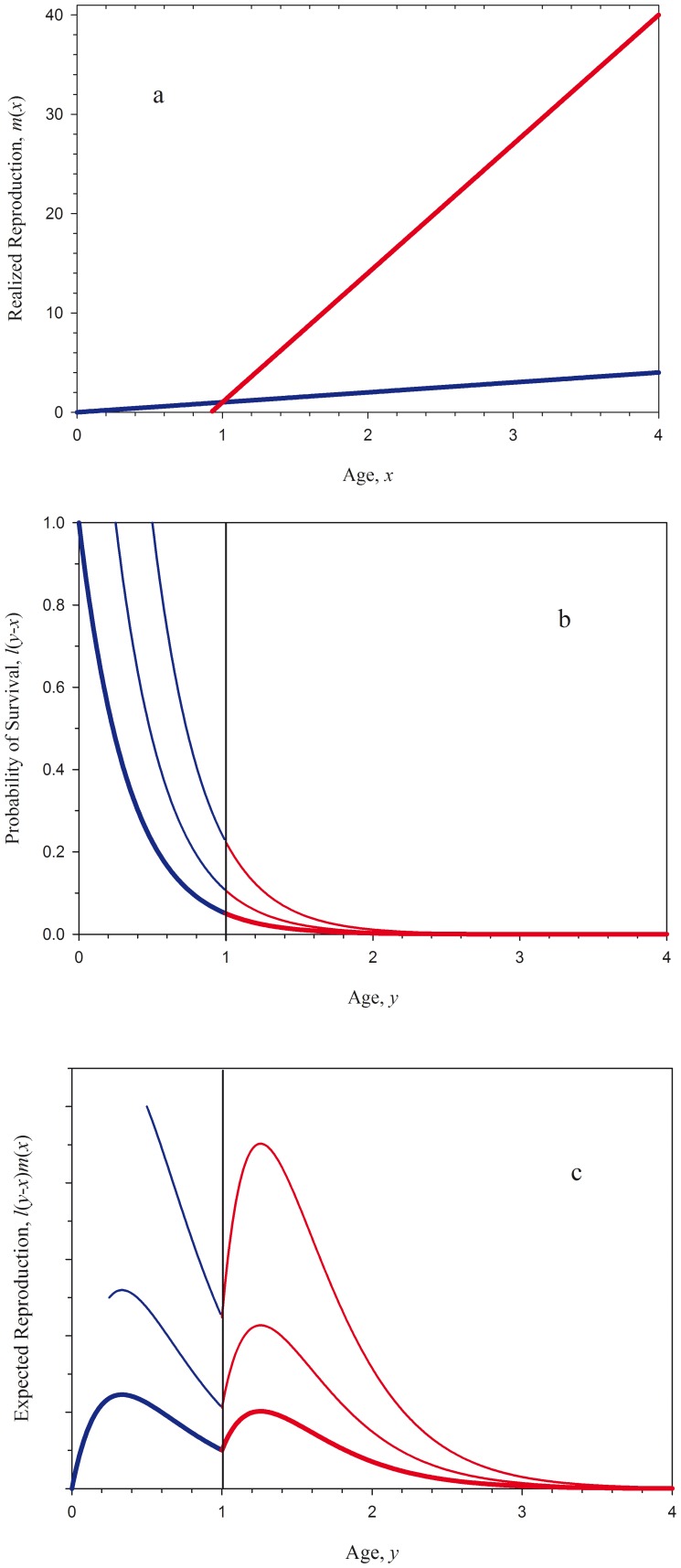
Changes in the age-specific distribution of reproductive value under sex change. a. Idealized male (red) and female (blue) reproductive rates as described in the classic interpretations of the size-advantage model [1]–[6]. The optimal size at sex change is at age 1, as such an individual has the higher reproductive rate at all ages. b. The probabilities of survival 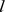
(*y*-*x*), for individuals of ages 0 (bold), 0.25, and 0.5. The remaining survival time is independent of current age (i.e., the survival function is said to be memoryless). In this example, an individual at age 0 has a 5% likelihood of reaching age 1, the age at sex change. c. The expected reproductive payoff, 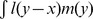, plotted against age. Figures 1a and 1b give rise to Leigh et al.'s [4] result in which half of the lifetime fitness comes through function in the initial sex (here, female), and half through function in the second sex (i.e. the areas under the blue and red curves are equal). As an individual ages, the expected fitness contribution through reproduction in the high-payoff second sex increases relative to expected contribution via the initial sex.

Sex change theory proposes that when size-specific reproduction increases at one rate for males and at another rate for females the functions describing these rates will inevitably cross, and sex change will be favored by natural selection [1]–[5]. This, Ghiselin's size-advantage hypothesis [1], simply and elegantly predicts both the direction and the timing of sex change. The direction of sex change is predicted by the relative reproductive rates: If small females have higher reproductive rates than small males, but large males have higher rates than large females, then protogyny, female-to-male sex change, is favored; if this relationship is reversed, then protandry, male-to-female sex change, is favored [1], [2]. The optimal timing of sex change is the age or size at which the reproductive rates cross [2]–[4] (Fig. 1a), holding the size-specific survival and growth rates equal across the sexes [5], [6].

In their seminal contribution to sex-change theory, Leigh et al. [4] make a crucial insight connecting the timing of sex change to Fisher's [7] explanation of a 1∶1 primary (i.e., at fertilization) sex ratio. Fisher observed that in sexually reproducing species total male fitness must be the same as total female fitness, as every individual has precisely one father and one mother. A consequence of this is that if sons cost twice as much as daughters, the equilibrium m∶f sex ratio will be 1∶2, as it is at this ratio that the fitness returns to a parent through either one son or two daughters are equal. When sons and daughters are equally costly, a seemingly common condition, particularly in species exhibiting external fertilization and no parental care, the primary sex ratio at equilibrium is 1∶1. Leigh et al. realized that Fisher's solution to the problem of optimal sex allocation across generations must also apply to a sequential hermaphrodite across its lifetime. Their analysis shows that the optimal age at sex change will be the age at which exactly half an individual's expected fitness will come through reproduction as a female and half through reproduction as a male.

### Incorporating variance in condition

Implicit to Leigh et al.'s solution is the assumption that individuals do not vary in condition. While individual condition or quality is often made tangible by measures such as body mass, nutritional state, experience, or genetic load [8], [9], the most coherent definition of condition is that offered by Rowe and Houle [10], who regard condition as simply that which positively correlates with future fitness.

Trivers and Willard [11] modified Fisher's model to propose that condition-dependence might influence the sex allocation of parents in species providing parental care. Specifically, they suggested that if an offspring's condition is correlated to that of its mother, and if condition affects male reproductive success (RS) more strongly than it affects female RS [12], [13], large, high-condition mothers would benefit from producing sons, while smaller, low-condition mothers would benefit from producing daughters (but see [14]). Substituting individual size across its lifetime for size of individuals within a population, we see it is the same relationship – male RS increasing at a higher rate than female RS, and thus exhibiting high variance over the lifetime – which drives both protogynous sex change [1] and selection on the sex ratio [11]. It is this parallel which motivates the current work.

A complication is that Trivers and Willard [11] argued that the effect of condition on the sex ratio would be limited to the individual, but did not believe that this effect would extend to the population. That is, they predicted that the over-production of sons by high-condition mothers would be balanced by an over-production of daughters by low-condition mothers, and that the population sex ratio would thus remain at (or very close to) 1∶1. The problem with Trivers and Willard's interpretation is that, if it is true that males have higher mean fitness than females, not simply higher variance in fitness, it must also be true that males are the rarer sex [7]. Thus, if the envisioned condition-dependence effect is operating, it will skew the population sex ratio [5], [15].

Here, as Trivers and Willard modified Fisher's analysis of sex ratio evolution, we modify that of Leigh et al. [4] to consider the effects of condition dependence on sex allocation in a sequential hermaphrodite. We find that condition dependence unbalances the fitness expectations arriving through the initial and second sexes. While this represents a shift in sex allocation, it also results in a shift in the optimal allocation of reproductive effort over an individual's lifetime.

## The Model

We develop an analytical model to solve for the optimal schedule of effort allocated to current versus future reproduction across the lifetime for a hypothetical species of protogynous sex changer based loosely on the biology of Labroid fishes. This formulation allows us to explore selection on sex allocation as a subcomponent of optimal life-history evolution. Our approach combines elements of Leigh et al.'s [4] population genetic and Williams' discrete time [18] models, but is more closely related to the graphical model developed by Pianka and Parker [16] and elaborated by Rogers and Sargent [27]. Here, we trace the evolution of our continuous-time model from its discrete-time foundation.

An optimal organism is one which maximizes its reproductive value, *V*, at every age [7], [17]–[20]. Under the assumption that reproduction takes place at discrete time intervals, this gives
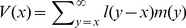
(eq.1)where *l*(*y-x*) is the probability of being alive at age *y* given that the individual is alive at *x*, and *m*(*y*) is the realized reproductive rate at *y*. Here, we assume that the survival rate does not decrease with age; that is, *l*(*y*-*x*) is said to be ‘memoryless’. Williams [18] separated reproductive value into two components, 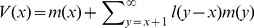
(eq.2)where the first term on the right-hand side represents the specific contribution via realized reproduction in the present, while the second term represents expected, realized reproduction in the future (what Williams [18] termed the residual reproductive value, *RRV*). The realized rates are themselves functions of the level of reproductive effort devoted to reproduction, *E*(*x*), or withheld as an investment in the future, 1 – *E*(*x*), where 0≤*E*(*x*)≤1. We shall assume that realized reproduction in the present can be represented as
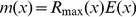
(eq.3)where *R*_max_(*x*) is the age-specific physiological maximum reproductive rate. An allocation of *E*(*x*) = 0 represents a juvenile phase at age *x*, and the value of future reproduction is maximized. Alternatively, when *E*(*x*) = 1, current reproduction is maximized while all future reproduction is sacrificed.

Williams [18] showed that the optimal level of reproductive effort at a given age, E^*^(*x*), is that which balances the marginal gain in present reproduction from a infinitesimal increase in *E* against the marginal loss in expected, future reproduction. For iteroparous species, the function describing the tradeoff between present and future reproduction must be concave-down (at least in the vicinity of the optimum), as linear or concave-up functions imply semelparity [16], [19], [21]. The optimal level of realized reproduction is the *x*-coordinate of the point on the curve whose tangent line has slope -1. We model the tradeoff with an ellipse with *x*-intercepts at ±*R*_max_(*x*) and *y*-intercepts at ±*RRV*_max_(*x*) as it represents a simple concave-down relationship between investments in present versus future reproduction. In this model, the optimal level of reproduction is 
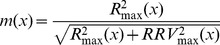
(eq.4)see Appendix S1. Thus, using the assumption in eq. 3, the optimal allocation, E^*^(*x*), is 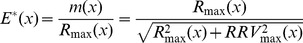
(eq.5)


If one is given the potential maximal reproductive rate at some arbitrary age *x*, *R*_max_(*x*), the potential maximal residual reproductive value, *RRV*_max_(*x*), and the function describing the tradeoff between current and future reproduction (e.g., eq.5), finding the optimal level of effort, E^*^(*x*), at any given age is straightforward; solving for the optimal schedule of allocation across the span of an individual's life, however, is not. Two factors complicate such a solution. The first is that, while the above potentials, *R*_max_(*x*) and *RRV*_max_(*x*), must exist, they are inherently unmeasureable. That is, a researcher can measure the realized reproductive rate, *m*(*x*), which she knows to be the product of potential and allocation, *R*_max_(*x*)×*E*(*x*), but the underlying values of *R*_max_(*x*) and *E*(*x*) cannot be directly measured. Nevertheless, it seems reasonable that one may infer the general form of *R*_max_(*x*) from *m*(*x*), and here we make this assumption. The age-/size-specific reproductive rate for a protogynous sex-changer is often modeled by a pair of intersecting functions: a linearly increasing function describing reproduction as a female, and an exponentially increasing function describing reproduction as a male (e.g., Figure 1a of [2]). While such a pair of functions captures the essence of the size-advantage model in the vicinity of the age/size at sex change, they are not mathematically realistic as they imply increases to infinity for both sexes. In reality, the potential reproductive rate of Labroid fishes is best described by a logistic function [22]. For simplicity, we here use a step-function,
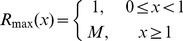
(eq.6)in place of the logistic to describe the age-specific maximal reproductive rate across both the female (*x*<1) and male (*x*≥1) segments of the life history in which male reproduction is *M* times that of a female (*M*>1). While this is a gross approximation of a logistic curve, particularly near the discontinuity, it does mimic the asymptotic behavior of such a curve while making the mathematics tractable.

Having dealt with the first complication, we turn to the second. Finding the level of allocation which maximizes *V*(*x*) requires that we know *RRV*_max_(*x*), which in turn requires that we know the optimal level of reproductive allocation at all future ages *y*>*x*. Such optimization problems are often solved numerically by the method of backwards iteration in a dynamic programming model [24]–[27]. In the current work we adopt a different method. We consider a system in which (i) individuals vary in condition, (ii) high-condition individuals have high survival rates, and (iii) the survival rate for an individual is independent of its age. This gives
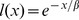
(eq.7)where *l*(*x*) is the probability of surviving to *x* from age 0 and *β* is the life expectancy which varies based on the condition of the individual. The simplified life history described by equations 6 and 7 allows us to approximate equation 5, and solve for the optimal life history analytically (see [28] for a similar approach).

If individuals within the population do not vary in condition, the m∶f sex ratio will be 
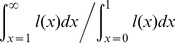
(eq.8)


As total male fitness must equal total female fitness [7], 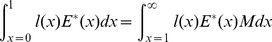
(eq.9)equation 9 gives rise to Leigh et al.'s [4] condition that half of an individual's expected fitness is fitness will come through reproduction as a female and the other half through reproduction as a male (Fig. 1c). It is interesting to note here that an individual's 1∶1 expectation changes as it ages. For individuals reaching ages>0, the expected contribution to fitness through reproduction in the high-payoff, second sex (here, male) increases relative to the expected contribution through the initial sex (female) for two reasons. First, as individuals grow older, an increasing fraction of initial-sex fitness has fallen in the past and thus no longer exerts an influence on the optimal allocation of effort. At the same time, the probability of reaching the high-payoff, second-sex phase of the life history has increased, increasing the reproductive value of this phase (Fig. 1c).

### Incorporating variance in condition - the effect on sex allocation

**Figure 2:**
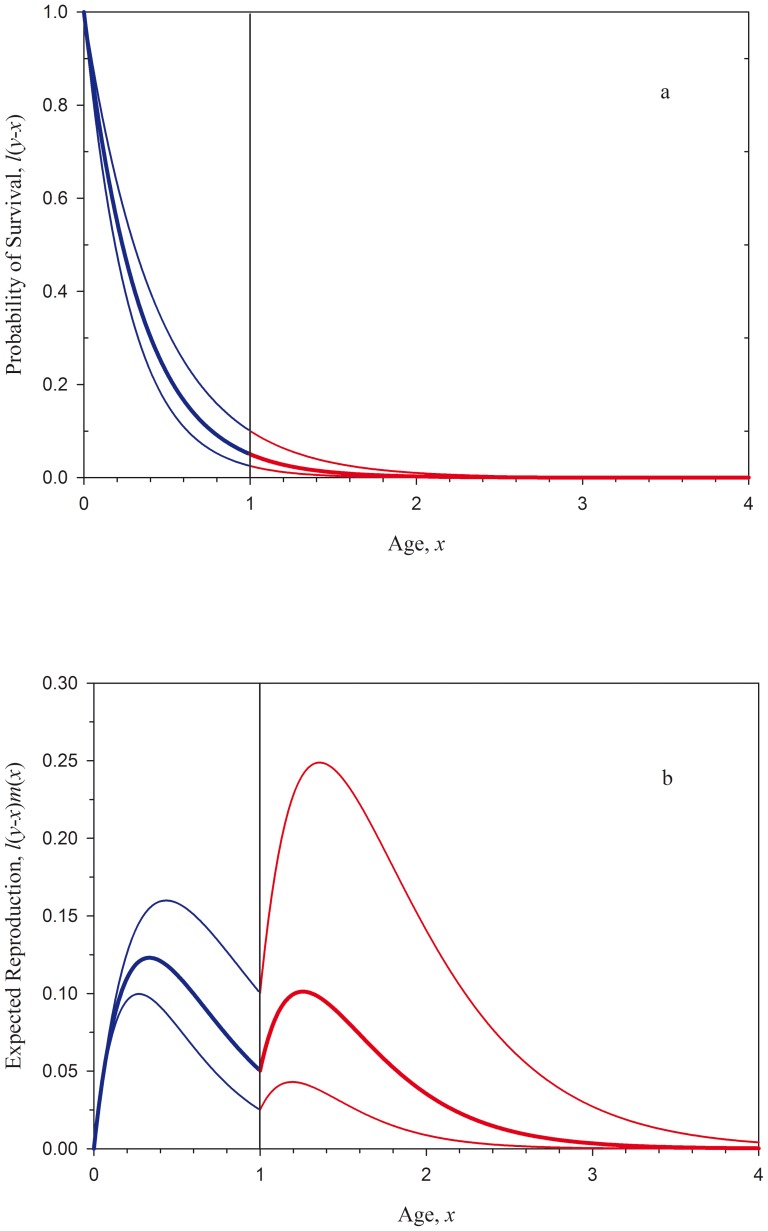
The effect of condition-dependent survival on the relative fitness contributions in the initial and second sexes. a. High- and low-condition individuals have higher and lower survival rates than an average individual (bold). In this example, high-condition individuals exhibit a 10% likelihood of reaching age 1, while those of low-condition exhibit only a 2.5% chance of reaching the second sex. b. For high-condition individuals, the fitness contribution (i.e., the area under the curve) from expected reproduction in the high-payoff second sex is much greater than the expected contribution in the initial sex; the opposite is true for individuals of low-condition. This is an application of Trivers and Willard's [11] modification of Fisher's [7] model of sex ratio evolution to a sequential hermaphrodite.

Given this, we now consider the fate of an individual of high condition within a population exhibiting variance in condition. As high-condition individuals have high survival rates, such individuals have higher reproductive value at all ages (eq.7 and eq.a14; see Appendix S2). However, the effect of the high survival rate on the initial- and second-sex components of the life history is not equal: the gains in expected fitness to a high-condition individual through the high-payoff second sex are greater than the gains in the initial sex (Fig. 2a). Likewise, an individual of low condition will have relatively low reproductive value across the lifetime, with the expected reproductive payoffs in the second sex again being disproportionately affected (Fig. 2b). Thus, as condition dependence is expected to alter the sex ratio of a parent's offspring in an outcrossing species [5], [15], it is similarly expected to alter the sex allocation of a hermaphrodite.

### Incorporating variance in condition - the effect on the allocation of reproductive effort

As noted above, we are here viewing sex allocation as a subcomponent of the optimal allocation of resources over the lifespan. To solve for the optimal schedule of allocation analytically, we must adapt the discrete-time interpretation of reproductive value (implied by equations 1 and 2 above) to a continuous-time interpretation. With *m*(*x*) defined in eq.3, the analogue of eq.2 is 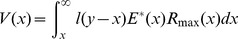
(eq.10)


Note that the division between current and future components of reproductive value vanishes in a continuous-time description of *V*. Since our survival rate function is memoryless, the reproductive value beyond age 1, the age at sex change, is a constant, *MC*, where *C* is 
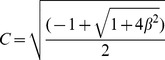
(eq.11)(see Appendix S2). For all ages *x*≥1, the reproductive value is the constant 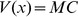
(eq.12)thus the realized reproductive rate is
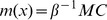
(eq.13)and the optimal allocation of reproductive effort is, by equation 5,
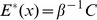
(eq.14)


On the other hand, E^*^(*x*) varies for ages *x*<1. The reproductive value across this phase of the life history is an increasing function satisfying 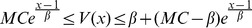
(eq.15)while the optimal level of effort, E^*^(*x*), is a decreasing function satisfying 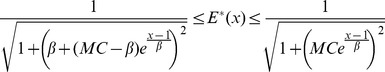
(eq.16)(see Appendix S3).

**Figure 3:**
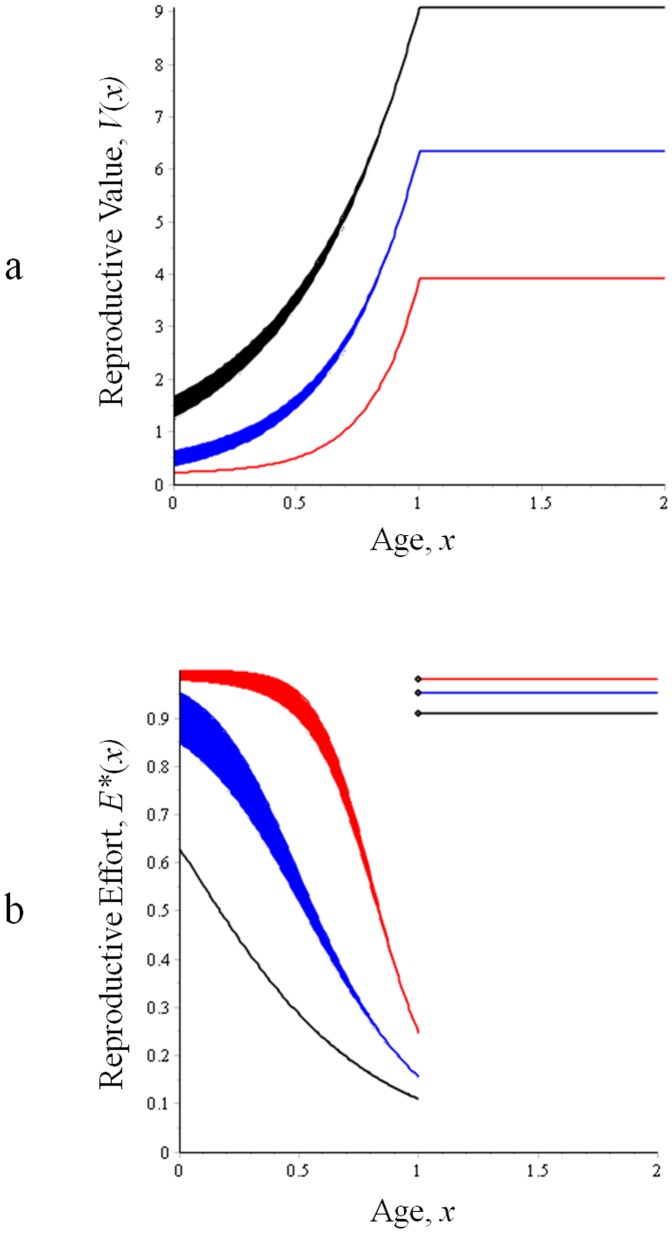
Condition-dependent effects on reproductive value and reproductive effort. a. Age-specific reproductive value for individuals of normal (blue), high (black), and low (red) condition. For any given age, the true value of the function lies within the limits of the thick lines; see text and Appendix S2. Reproductive value increases as individuals approach age 1, the age at sex change. b. The optimal allocation of reproductive effort, E^*^(*x*) between age 0 and age 1 for individuals of high, normal, and low condition. Allocation to current reproduction decreases as future, expected reproduction increases in value with the approach to the age at sex change [27]. Low-condition individuals exhibit increased allocations to current reproduction, as the value of future reproduction is reduced by low survival rates.

Recalling that the survival rate, and thus the lifespan, *β*, is a function of condition, we see from eq.16 that high-condition individuals are predicted to have lower allocations of reproductive effort at all ages, while having higher lifetime fitness, than lower condition individuals (eq.15; Fig. 3). This leads to the somewhat counterintuitive result that a low-condition female may produce more eggs than does a high-condition female in a given reproductive bout [16]–[18] (but see [27]).

## Discussion

Eighty years ago, Fisher [7] explained why the sex ratio in an outbreeding species without post-zygotic parental care may be expected to be 1∶1. Extending Fisher's logic to sequential hermaphrodites, Leigh et al. [4] showed that the optimal timing of sex change will be the age or size at which half of an individual's expected reproductive success arrives through function as a female, and half through function as a male. However, these results [4], [7] hold only if the individuals within the population have equal fitness expectations; that is, if they are equal in condition (sensu [10]). Trivers and Willard [11] relaxed this assumption to consider the effects of condition dependence on the evolution of the sex ratio. High-condition individuals may be larger, have greater fat reserves, fewer parasites, or control a territory richer than average. The specifics of what endows an individual with ‘high condition’ are immaterial – what is important is that such individuals have higher than average fitness expectations [10]. Trivers and Willard predicted that if there was a correlation between the condition of a parent and that of its offspring, natural selection would favor an over-production of offspring in the sex exhibiting higher variance in reproductive success as an adult by high-condition parents [11]–[14], [29], [30].

Here, we apply the Trivers-Willard argument to Leigh et al.'s analysis in order to investigate the effects of condition dependence on both sex allocation and the optimal schedule of reproductive effort across the lifetime in a sequential hermaphrodite. To do so, we model an age-specific life history in which the survival rate varies with individual condition, but the growth rate is constant. If we were to regard *x* to instead represent size, ours becomes a size-specific model in which the growth rate varies as a function of condition and the mortality rate is constant. Doing this would effectively make fecundity condition-dependent. Under either interpretation our results remain the same – the key variable is that high-condition individuals have higher fitness expectations than individuals of average (or low) condition, and making survival or growth condition-dependent is a simple way of achieving this.

In contrast to Leigh et al.'s [4] result, we find that individual condition is expected to unbalance the reproductive values of the initial and second sexes (Fig. 2). For example, if an individual of average condition exhibits a 1∶1 ratio of expected reproductive value in the initial and second sexes, a high-condition individual might exhibit a ratio of 1∶2. To be more specific, consider the biology of Labroid fishes. Protogynous sex change is common to the fishes in the suborder Labroidea, and in some species, for example the well-studied bluehead wrasse (*Thalassoma bifasciatum*), the reproductive rate of a sex-changed male can be an order of magnitude higher than that of an initial sex female [31], [32]. In spite of this, under Leigh et al. [4], the expected fitness payoffs accumulating over the female and the male phases of the life history should be equal, as the high reproductive rate of a male is offset by the relatively low probability of surviving long enough to become one [22], [23]. Our model shows that while variance in condition affects the expected reproductive value in both the initial and the second sexes, the effect on the high-payoff second sex is much greater (Fig. 2). Individuals with above- or below-average levels of condition are thus predicted to experience unequal fitness payoffs between the initial and second sexes. This condition-dependent skew in the sex allocation of an individual across its lifetime is analogous to the condition-dependent skew in a parent's sex ratio envisioned by Trivers and Willard [11].

Because of its effect on sex allocation, individual condition is also predicted to alter the optimal schedule of reproductive effort in such systems. As Williams [17], [18] has shown, the optimal allocation of reproductive effort is that which balances the payoff between reproduction in the current bout and expected reproduction in the future (see also [16], [19], [20], [27]). For a young, small individual, the optimal allocation is usually a very slight investment in present reproduction, as most of the individual's reproductive value is lies in its future. In contrast, an old individual may have a very low expectation of future reproduction, and would maximize its fitness by investing more fully in reproduction in the current bout. For organisms experiencing senescence, this results in a monotonic increase in the allocation of reproductive effort across the lifetime [16], [17], [27]; this expectation is known as Williams' Principle. As a counterexample to this principle, Rogers and Sargent [27] showed that the dramatic increase in an individual's reproductive rate upon sex change in a species like *T. bifasciatum* can cause the gains in residual reproductive value to outpace the gains in current reproduction, favoring a decrease in the allocation of effort with increasing size. Our model here confirms Rogers and Sargent's prediction: For an individual of average condition at age 0, the reproductive value coming to an individual early in its life as a female precisely equals that expected to come late in life as a male ([4]; see Appendix S3). However, as an individual ages, the very fact of its survival to any age>0 shifts this expectation (Fig. 1b), and the optimal allocation of effort declines with the approach to the age at sex change (Fig. 3b; This effect is made visible by our use of a step function to describe the potential reproductive rate). Condition acts to affect the allocation of effort in a similar way. As an individual of high condition has an increased expectation of reproduction in the high-payoff second sex, it is predicted to exhibit a reduced allocation to reproduction in the initial sex relative to an average individual (Fig. 3b). Correspondingly, low-condition individuals are expected to have an increased allocation to early-life reproduction as their expectation of reproduction later in life is reduced. While it might be challenging to test in nature, this leads to the prediction that fish with the highest lifetime fitness expectations might reproduce at a lower rate, albeit over a longer lifespan, relative to low-condition fish.

## Supporting Information

Appendix S1 **Computing the optimal realized reproduction.**
(DOCX)

Appendix S2 **Modeling the behavior after sex change.**
(DOCX)

Appendix S3 **Modeling the behavior before sex change.**
(DOCX)
