# Supplementary material for: Condition Dependent Effects on Sex Allocation and Reproductive Effort in Sequential Hermaphrodites
Source: PLoS One. 2014 Oct 10;9(10):e109626. doi: 10.1371/journal.pone.0109626 (PMC4193790; doi:10.1371/journal.pone.0109626)
Supplement: Appendix S1 — Computing the optimal realized reproduction. (DOCX) [file pone.0109626.s001.docx]

*Appendix S1: Computing the optimal realized reproduction*

As stated in the text, Williams [18] showed that the optimal level of reproductive effort at a given age, *E^*^*(*x*), is that which balances the marginal gain in present reproduction from a infinitesimal increase in *E* against the marginal loss in expected, future reproduction. In a continuous model, the optimal value of *E* is the point where

 $m\left( x \right)=R_{max}(x,E(x))$ . eq.a1

By the chain rule for derivatives,

 $m\left( x \right)=R_{max}(x,E(x))$ , eq.a2

and combining the two equations above gives

 $m\left( x \right)=R_{max}(x,E(x))$ . eq.a3

Thus, to determine the optimal value of *E* we need to find the point on the curve describing the tradeoff between present and future reproduction whose tangent line has slope 1.

To simplify notation, consider an ellipse with *x*-intercepts at ±*a* and *y*-intercepts at ±*b*:

 $m\left( x \right)=R_{max}(x,E(x))$ . eq.a4

We will determine where slope of the tangent line to this curve is precisely *dy*/*dx* = -1 for *x*, *y* > 0

 $m\left( x \right)=R_{max}(x,E(x))$ , eq.a5

 $m\left( x \right)=R_{max}(x,E(x))$ , eq.a6

 $m\left( x \right)=R_{max}(x,E(x))$ , eq.a7

 $m\left( x \right)=R_{max}(x,E(x))$ . eq.a8

By substituting this into the ellipse equation and solving for *x* we get

 $m\left( x \right)=R_{max}(x,E(x))$ , eq.a9

 $m\left( x \right)=R_{max}(x,E(x))$ , eq.a10

 $m\left( x \right)=R_{max}(x,E(x))$ , eq.a11

 $m\left( x \right)=R_{max}(x,E(x))$ . eq.a12

Replacing *a* with *R*_max_(*x*), *b* with *RRV_max_*(*x*), and *x* with *m*(*x*) yields

**** $m\left( x \right)=R_{max}(x,E(x))$ . eq.a13
