# Supplementary material for: Condition Dependent Effects on Sex Allocation and Reproductive Effort in Sequential Hermaphrodites
Source: PLoS One. 2014 Oct 10;9(10):e109626. doi: 10.1371/journal.pone.0109626 (PMC4193790; doi:10.1371/journal.pone.0109626)
Supplement: Appendix S2 — Modeling the behavior after sex change. (DOCX) [file pone.0109626.s002.docx]

*Appendix S2: Modeling the behavior after sex change*

We start with the continuous version of eq.1. Since the reproductive value *V*, is constant for *x* > 1 and depends on *M* we write *V* = *MC* for some constant *C*. Here, we determine the value of *C*. First, notice

$V\left( x \right)=\int_{x}^{\infty} l\left( y-x \right)m(y)dy=\int_{x}^{\infty} {exp}^{-(y-x)/\beta}m(y)dy={exp}^{x/\beta}\int_{x}^{\infty} {exp}^{-y/\beta}m(y)dy$ . eq.a14

If we differentiate both sides with respect to *x* we get

$m\left( x \right)=R_{max}(x,E(x))$ . eq.a15

$V^{'}\left( x \right)=-{exp}^{\frac{x}{\beta}}{exp}^{-\frac{x}{\beta}}m\left( x \right)+\beta^{-1}{exp}^{\frac{x}{\beta}}\int_{x}^{\infty} {exp}^{-\frac{y}{\beta}}m\left( y \right)dy=-m\left( x \right)+\beta^{-1}V\left( x \right)$As *V*(x) is constant we get $m(x)=\beta^{-1}V(x)=\beta^{-1}MC$. Combining this with

$V(0)=MC{exp}^{- \frac{1}{\beta}}$ ,

$$\boldsymbol{m}\left( \boldsymbol{x} \right)\boldsymbol{=}\frac{\boldsymbol{R}_{\boldsymbol{max}}^{\boldsymbol{2}}\boldsymbol{(x)}}{\sqrt{\boldsymbol{R}_{\boldsymbol{max}}^{\boldsymbol{2}}\left( \boldsymbol{x} \right)\boldsymbol{+}\boldsymbol{RRV}_{\boldsymbol{max}}^{\boldsymbol{2}}\boldsymbol{(x)}}}$$

$$\boldsymbol{m}\left( \boldsymbol{x} \right)\boldsymbol{=}\frac{\boldsymbol{R}_{\boldsymbol{max}}^{\boldsymbol{2}}\boldsymbol{(x)}}{\sqrt{\boldsymbol{,,}\begin{aligned} \\ \\ \\ \\ \\ \\ \begin{aligned} \\ \\ \boldsymbol{R}_{\boldsymbol{max}}^{\boldsymbol{2}}\left( \boldsymbol{x} \right)\boldsymbol{+}\boldsymbol{RRV}_{\boldsymbol{max}}^{\boldsymbol{2}}\boldsymbol{(x)} \end{aligned} \end{aligned}}}$$

equation 4 from the text, gives

$\beta^{-1}MC=\frac{M^{2}}{\sqrt{M^{2}+{M^{2}C}^{2}}}=\frac{M}{\sqrt{1+C^{2}}}$. eq.a16

Simplifying eq.a16 gives

$\beta^{-1}C=\frac{1}{\sqrt{1+C^{2}}}$ , eq.a17

and solving this for *C* gives eq.11 (see text).
