# Supplementary material for: Condition Dependent Effects on Sex Allocation and Reproductive Effort in Sequential Hermaphrodites
Source: PLoS One. 2014 Oct 10;9(10):e109626. doi: 10.1371/journal.pone.0109626 (PMC4193790; doi:10.1371/journal.pone.0109626)
Supplement: Appendix S3 — Modeling the behavior before sex change. (DOCX) [file pone.0109626.s003.docx]

*Appendix S3: Modeling the behavior before sex change*

Eq.a15 (Appendix S2) above holds for any *x* > 1. If we assume *x* < 1, then we may use eq.4 to rewrite the equation as

$V^{'}=\beta^{-1}V-\frac{1}{\sqrt{1+V^{2}}}$ , eq.a18

with the initial value *V*(1) = *MC.* This differential equation has *V*(*x*) = *C* as its only positive equilibrium solution. Since *V*(1) = *MC* > *C* we know that *V*(*x*) is an increasing function for 0 < *x* < 1. The quotient on the right of eq.a18 clearly takes values between 0 and 1: actually,

$\frac{1}{\sqrt{1+C^{2}}}<\frac{1}{\sqrt{1+V^{2}}}<1$ $m\left( x \right)=\beta^{-1}MC$ , eq.a19

but for simplicity we will take 0 for the lower bound. Thus,

$\beta^{-1}V-1\leq V^{'}\leq\beta^{-1}V$ $m\left( x \right)=\beta^{-1}MC$ . eq.a20

We let *V_L_*(*x*) and *V_U_*(*x*) denote the functions which solve the initial value problems on the lower and upper bounds for *V*’ given above. Then

$V_{L}^{'}=\beta^{-1}V_{L}\to V_{L}\left( x \right)=V_{L}(0)e^{\frac{x}{\beta}}$ $m\left( x \right)=\beta^{-1}MC$ , eq.a21

and

$V_{U}^{'}=\beta^{-1}V_{U}-1\to V_{U}\left( x \right)=\beta+\left( V_{L}\left( 0 \right)-\beta\right)e^{\frac{x}{\beta}}$ $m\left( x \right)=\beta^{-1}MC$ , eq.a22

where *V*(0) is constant. Since *V_L_*(1) = *V_U_*(1) = *MC*, we obtain

 $m\left( x \right)=\beta^{-1}MC$ , eq.a23

and

 $m\left( x \right)=\beta^{-1}MC$ . eq.a24

Therefore, for *x* < 1 we have

 $m\left( x \right)=\beta^{-1}MC$ . eq.a25

Using equation 5, this allows us to construct bounds for *E*^*^(*x*) *= m(x)*: $m\left( x \right)=\beta^{-1}MC$ . eq.a26
